# Atheroembolic kidney disease: The under‐recognized silent killer

**DOI:** 10.1002/ccr3.3874

**Published:** 2021-01-28

**Authors:** Gabriel Ștefan, Adrian Zugravu, Simona Stancu, Mihaela Gherghiceanu, George Terinte‐Balcan

**Affiliations:** ^1^ “Dr. Carol Davila” Teaching Hospital of Nephrology Bucharest Romania; ^2^ Department of Nephrology “Carol Davila” University of Medicine and Pharmacy Bucharest Romania; ^3^ Department of Cell Biology and Histology “Carol Davila” University of Medicine and Pharmacy Bucharest Romania; ^4^ Ultrastructural Pathology “Victor Babes” National Institute of Pathology Bucharest Romania

**Keywords:** atheroembolic kidney disease, blue toe, cholesterol crystals, livedo reticularis, systemic atherosclerosis

## Abstract

While kidney biopsy demonstrating cholesterol crystal emboli is the method of definitive diagnosis; the triad of acute to subacute renal failure with skin findings in the setting of recent precipitating event should raise clinical suspicion for atheroembolic kidney disease.

## Graphical abstract

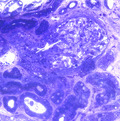

Atheroembolic disease is an under‐recognized cause of kidney failure. The triad of acute to subacute renal failure with skin findings in the setting of recent precipitating event should raise clinical suspicion. We report a rare finding with cholesterol crystals lodged in the afferent arteriole of the glomerulus after coronary angiography.

A 69‐year‐old male patient with hypertension was diagnosed to have anterior wall myocardial infarction and coronary angiography was performed. Occlusion of the proximal left anterior descending coronary artery (LAD) was documented and balloon angioplasty with stent placement in the proximal and mid LAD were done with drug‐eluting stent.

Four weeks after the coronary intervention, the patient was reassessed in our clinic with progressive loss of kidney function (serum creatinine 1.4‐6.9 mg/dL), increasing proteinuria (1.9 g/day) and hyperkalemia (6.6 mmol/L). Clinical examination demonstrated livedo reticularis with incipient necrotic spots on the thigh skin and blue toe syndrome with palpable distal pulses in both extremities (Figure 1). We performed kidney biopsy which identified cholesterol crystal emboli in afferent arteriole (Figure 2). Abdominal ultrasound detected ulcerated atherosclerotic plaque on the descending aorta.

**FIGURE 1:**
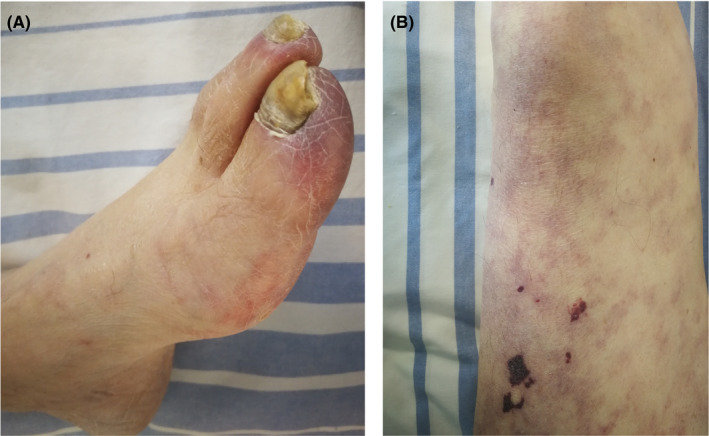
A, Blue toe syndrome; B, Livedo reticularis on the thigh skin

**FIGURE 2:**
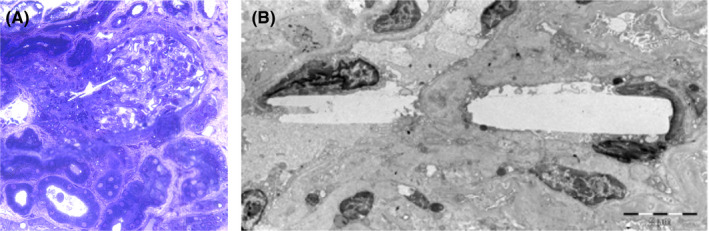
A, Light microscopy showing a glomerulus with a cholesterol crystal lodged in the afferent arteriole (semithin sections of Epon‐embedded kidney biopsy tissue colored with toluidine blue, 100×); B, Low magnification transmission electron image of a cholesterol crystal wedged in the glomerular vascular pole

The patient was initiated in hemodialysis on a tunneled jugular catheter. Also, treatment with statins and low dose corticosteroids (0.3 mg/Kg) was started. Unfortunately, at 3 months, the patient was still on renal replacement therapy.

Coronary angiography is the most frequent iatrogenic procedure causing embolism, with a reported incidence between 0.06% and 1.8%.^1^ The triad of a precipitating event (ie, vascular surgery, catheterization, anticoagulation, or thrombolysis), acute or subacute renal failure, and skin lesions, is strongly suggestive of atheroembolic kidney disease.^1^, ^2^ However, spontaneous event induced by hemodynamic stress has been reported in up to 30% of cases.^2^

Kidney biopsy is the definitive method for diagnosis, but is not necessary in patients with iatrogenic atheroembolic kidney disease presenting with the clinical triad.^1^ Cholesterol crystal emboli are identified in the lumen of arcuate and interlobular arteries, rarely, small crystals lodge in the afferent arterioles and glomerular capillaries.^1^

Treatment is mostly preventive, based on avoidance of precipitating factors. Statins, which stabilize atherosclerotic plaques, should be prescribed to all patients. Due to the inflammatory response, steroids might have a role in acute or subacute progressive forms.^1^ However, the outcome is poor with 40% renal survival and 17‐81% patient survival at 1 year.^1^

Atheroembolic disease is an under‐recognized cause of kidney failure and it should be included in the differential diagnosis of any elderly patient with progressive kidney failure and systemic atherosclerosis.

## CONFLICT OF INTEREST

None declared.

## AUTHOR CONTRIBUTIONS

Gabriel Ștefan, George Terinte‐Balcan: wrote the initial draft and revised the manuscript for critically important intellectual content; Adrian Zugravu, Simona Stancu, Mihaela Gherghiceanu: revised the manuscript and approved the final form, assisted in procuring and editing the images.

## INFORMED CONSENT

The patient provided written informed consent for the publication of the images and clinical data.
